# Supplementary material for: Lung, Breast and Colorectal Cancer Incidence by Socioeconomic Status in Spain: A Population-Based Multilevel Study
Source: Cancers (Basel). 2021 Jun 5;13(11):2820. doi: 10.3390/cancers13112820 (PMC8201149; doi:10.3390/cancers13112820)
Supplement: Supplementary file 1 [file cancers-13-02820-s001.zip › Supplementary_Files.pdf]

Supplementary Files

Supplementary Figure S1. Map of peninsular Spain with the provinces of the study highlighted by anatomical site: (a) Colorectum, (b) Lung, (c) Breast.

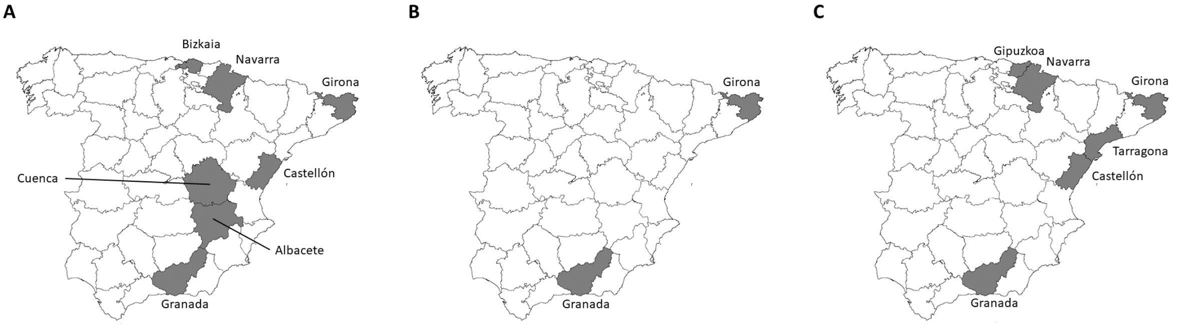

Supplementary Figure S2. Spanish deprivation index (2011) and average income (in €) per person (2015) by census tract in Spain, with generalized additive model (GAM) smoothing.

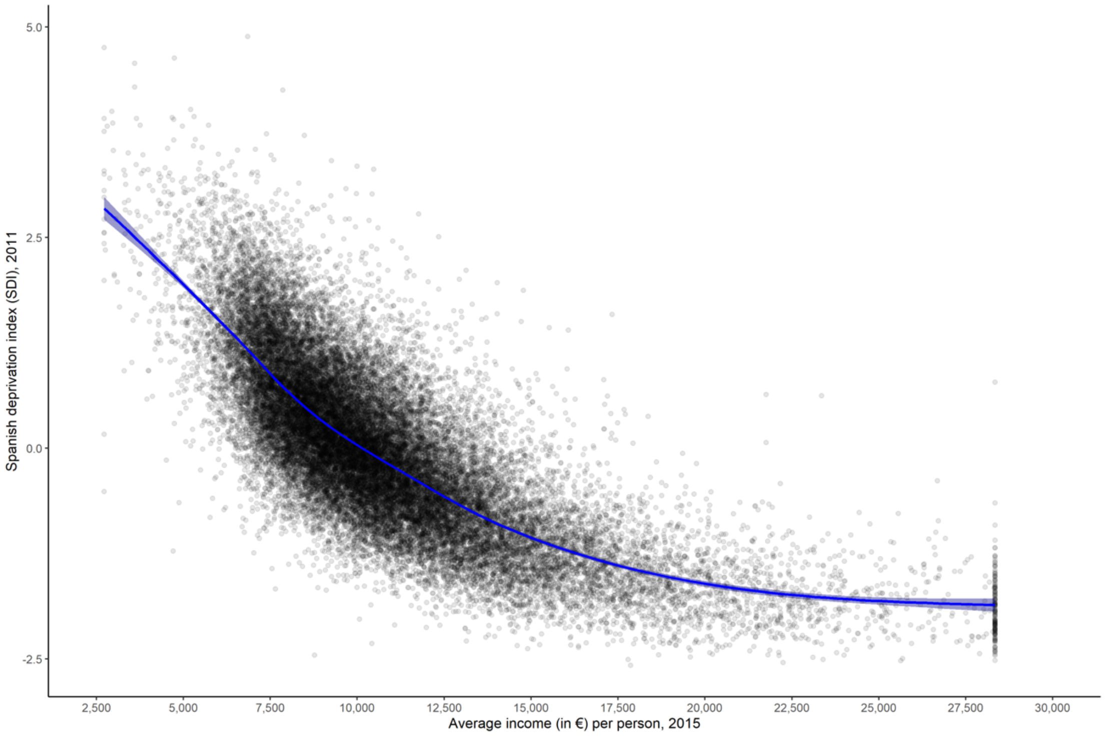

Legend: The range of average income was censured for confidentiality reasons.

Supplementary Figure S3. Quintiles of the Spanish Deprivation Index (SDI) by census tract in the capitals of the nine Spanish provinces of study, 2011.

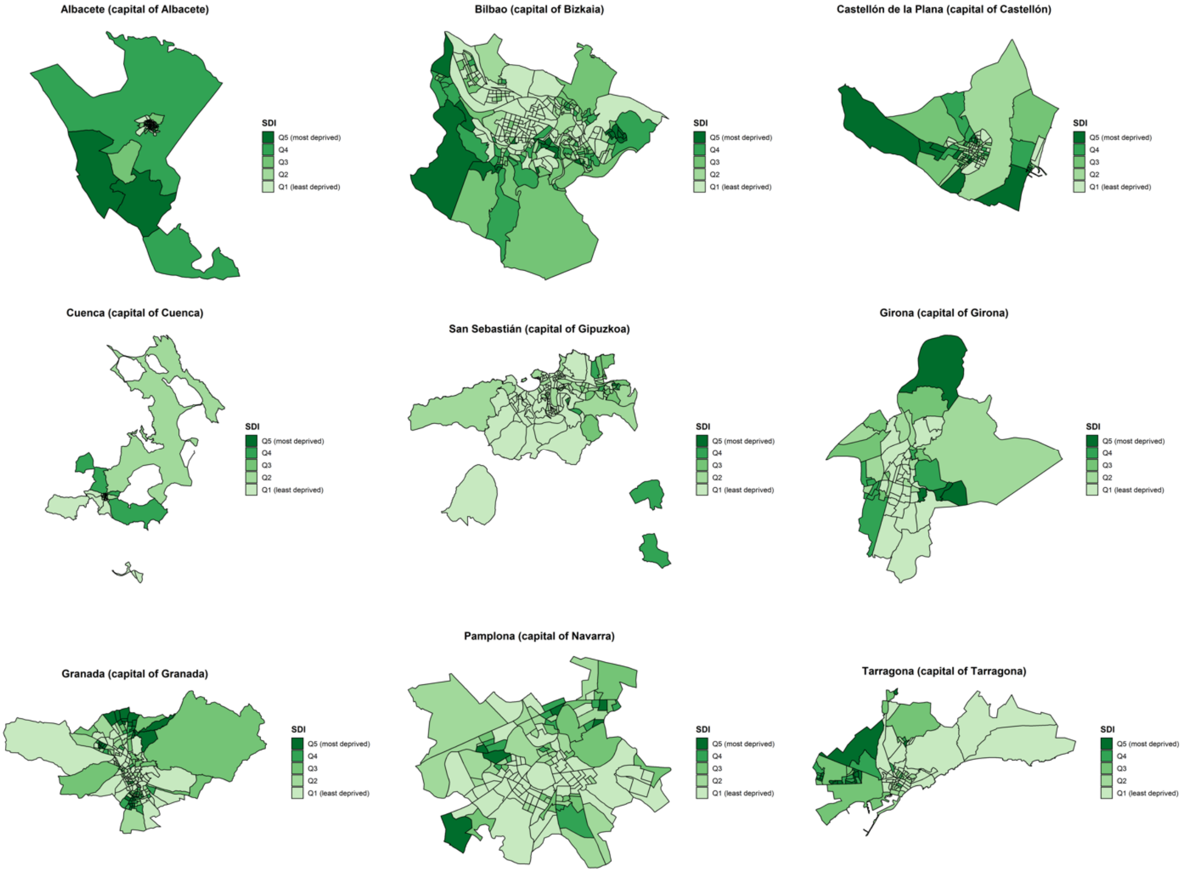

Supplementary Figure S4. Colorectal cancer smoothed cancer incidence rates adjusted for deprivation and age by census tract in the area of the capital of province in seven Spanish provinces during 2010-2013.

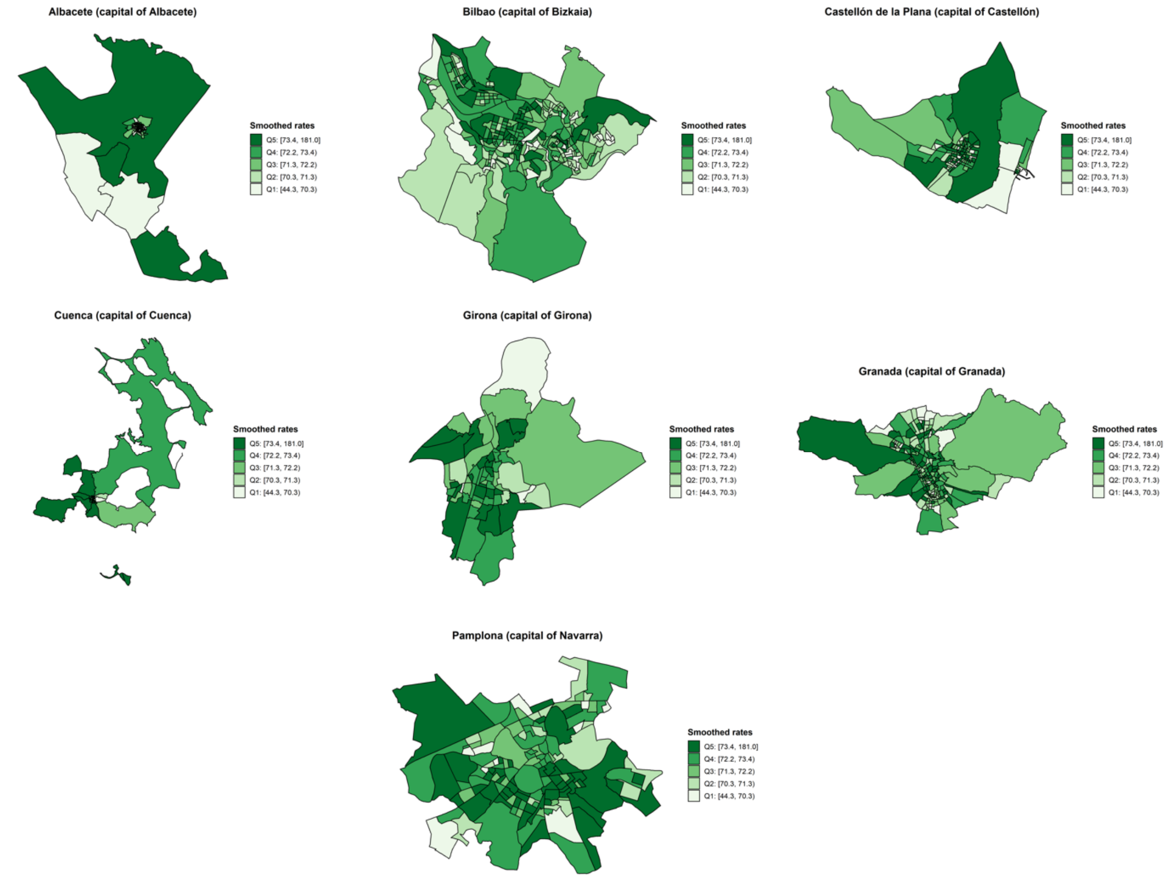

Supplementary Figure S5. Lung cancer smoothed cancer incidence rates adjusted for deprivation and age by census tract in the area of the capital of province in two Spanish provinces during 2010-2013.

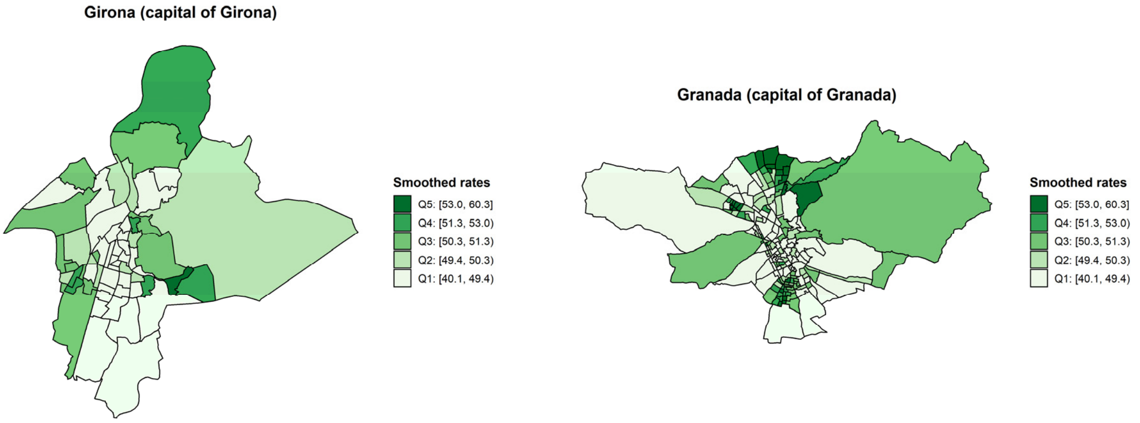

Supplementary Figure S6. Breast cancer smoothed cancer incidence rates adjusted for deprivation and age by census tract in the area of the capital of province in six Spanish provinces during 2010-2013.

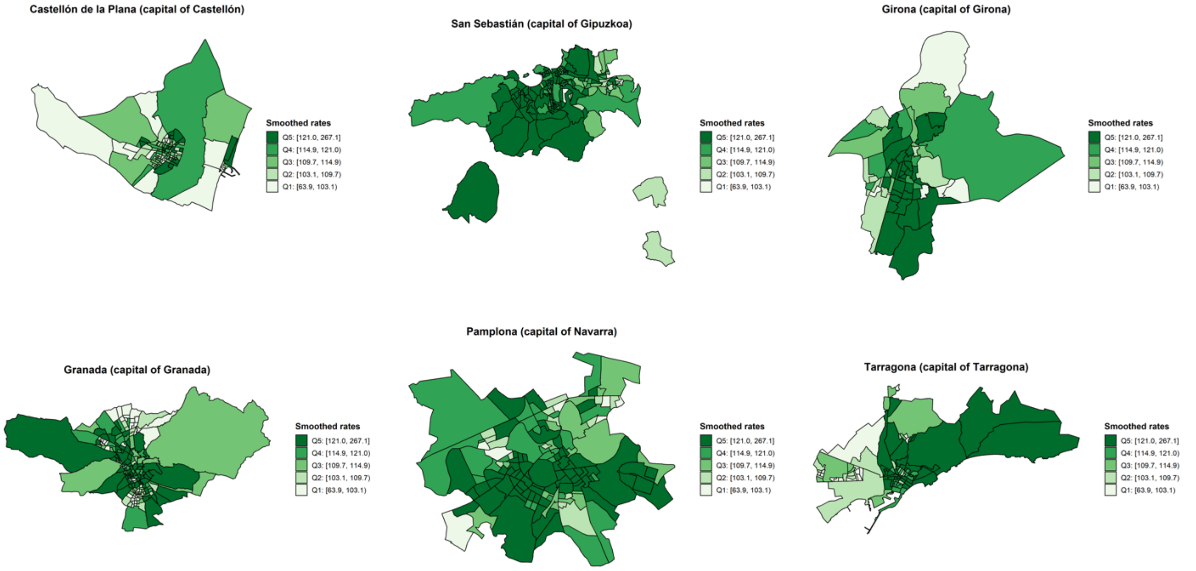

Supplementary Table S1. Number of colorectal cancer cases, population at risk, and crude cancer incidence rates by year of diagnosis, and province in Spain during 2010-2013.

Province	Cases	Population	Incidence rate* (95%CI)
Albacete 2011	217	402,318	53.9 (47.0–61.6)
Albacete 2012	185	402,837	45.9 (39.5–53.0)
Bizkaia 2011	633	1,155,727	54.8 (50.6–59.2)
Castellón 2011	412	604,344	68.2 (61.8–75.1)
Castellón 2012	398	604,564	65.8 (59.5–72.6)
Cuenca 2010	137	217,716	62.9 (52.8–74.4)
Cuenca 2011	171	219,138	78.0 (66.8–90.7)
Cuenca 2012	146	218,036	67.0 (56.6–78.8)
Girona 2011	497	756,810	65.7 (60.0–71.7)
Granada 2011	540	924,499	58.4 (53.6–63.6)
Navarra 2013	487	641,129	76.0 (69.4–83.0)

* Per 100,000 people

Supplementary Table S2. Number of lung cancer cases, population at risk, and crude cancer incidence rates by anatomical site, year of diagnosis, and province in Spain during 2011-2012.

Province	Cases	Population	Incidence rate* (95%CI)
Girona 2011	310	756,744	41.0 (36.5–45.8)
Granada 2011	375	924,550	40.6 (36.6–44.9)
Granada 2012	382	922,837	41.4 (37.4–45.8)

* Per 100,000 people

Supplementary Table S3. Number of breast cancer cases, population at risk, and crude cancer incidence rates by anatomical site, year of diagnosis, and province in Spain during 2010-2013.

Province	Cases	Population	Incidence rate* (95%CI)
Castellón 2011	302	301,462	100.2 (89.2–112.1)
Castellón 2012	302	301,406	100.2 (89.2–112.2)
Girona 2010	365	371,722	98.2 (88.4–108.8)
Girona 2011	406	375,324	108.2 (97.9–119.2)
Gipuzkoa 2011	482	361,706	133.3 (121.6–145.0)
Granada 2011	499	467,365	106.8 (97.6–116.6)
Navarra 2013	443	321,814	137.7 (125.1–151.1)
Tarragona 2011	358	401,669	89.1 (80.1–98.9)

* Per 100,000 people
