# Supplementary figures and images for: Lung, Breast and Colorectal Cancer Incidence by Socioeconomic Status in Spain: A Population-Based Multilevel Study
Source: Cancers (Basel). 2021 Jun 5;13(11):2820. doi: 10.3390/cancers13112820 (PMC8201149; doi:10.3390/cancers13112820)

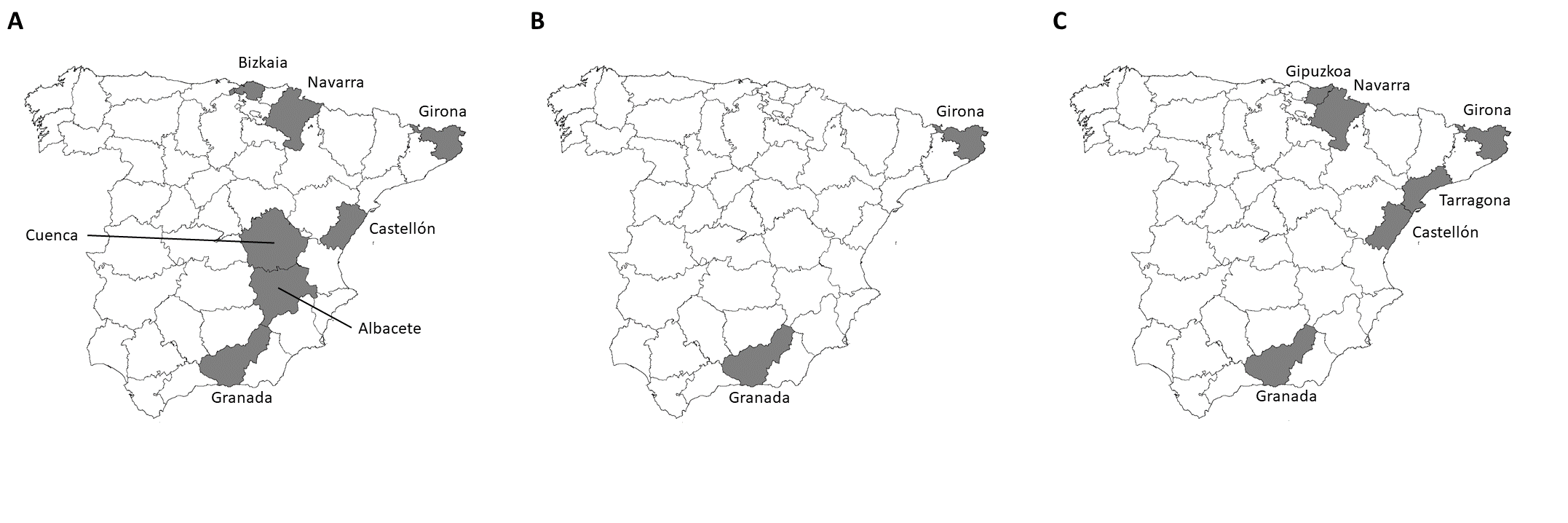

Supplement: Supplementary file 1 [file cancers-13-02820-s001.zip › SupFigure1.png]

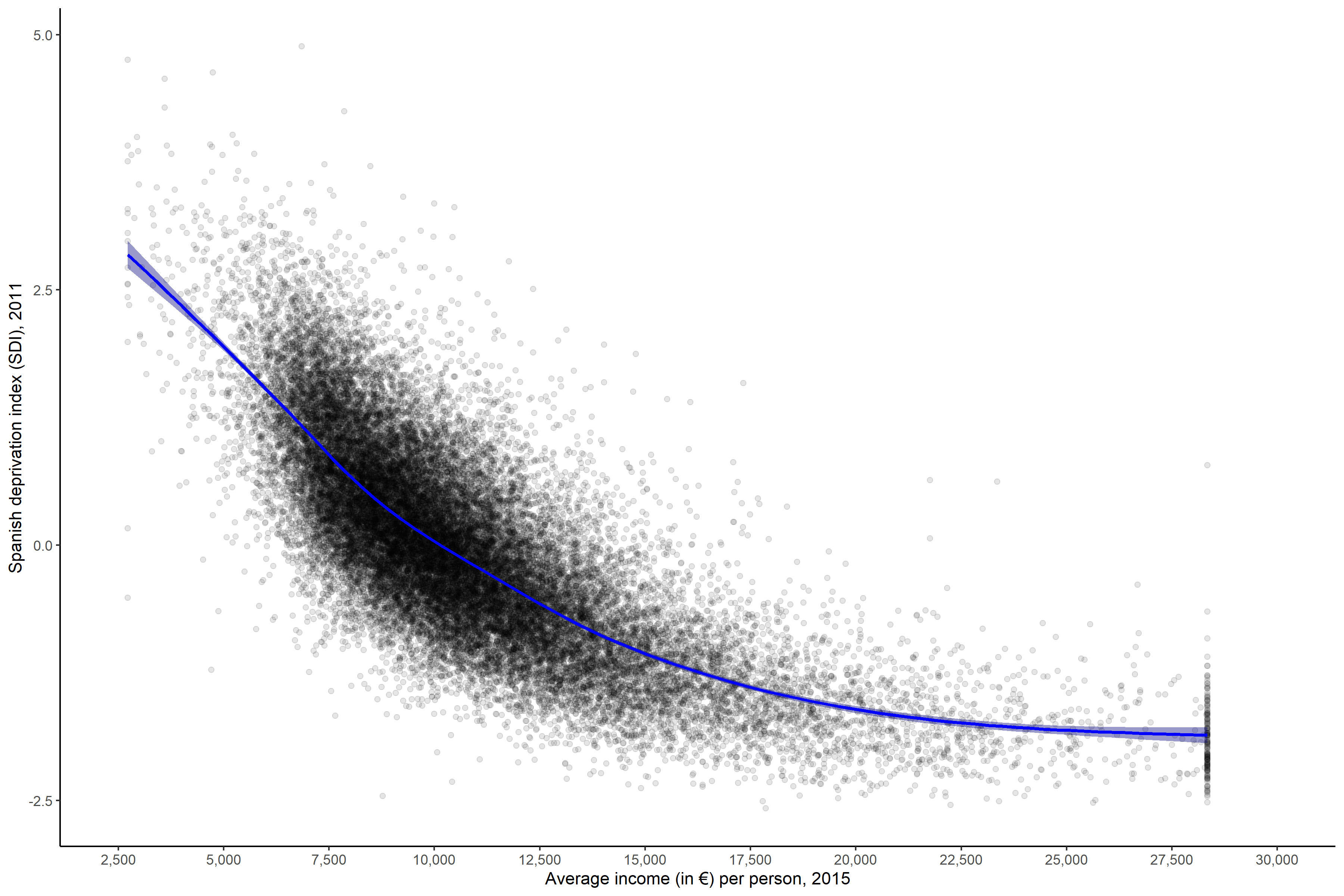

Supplement: Supplementary file 1 [file cancers-13-02820-s001.zip › SupFigure2.png]

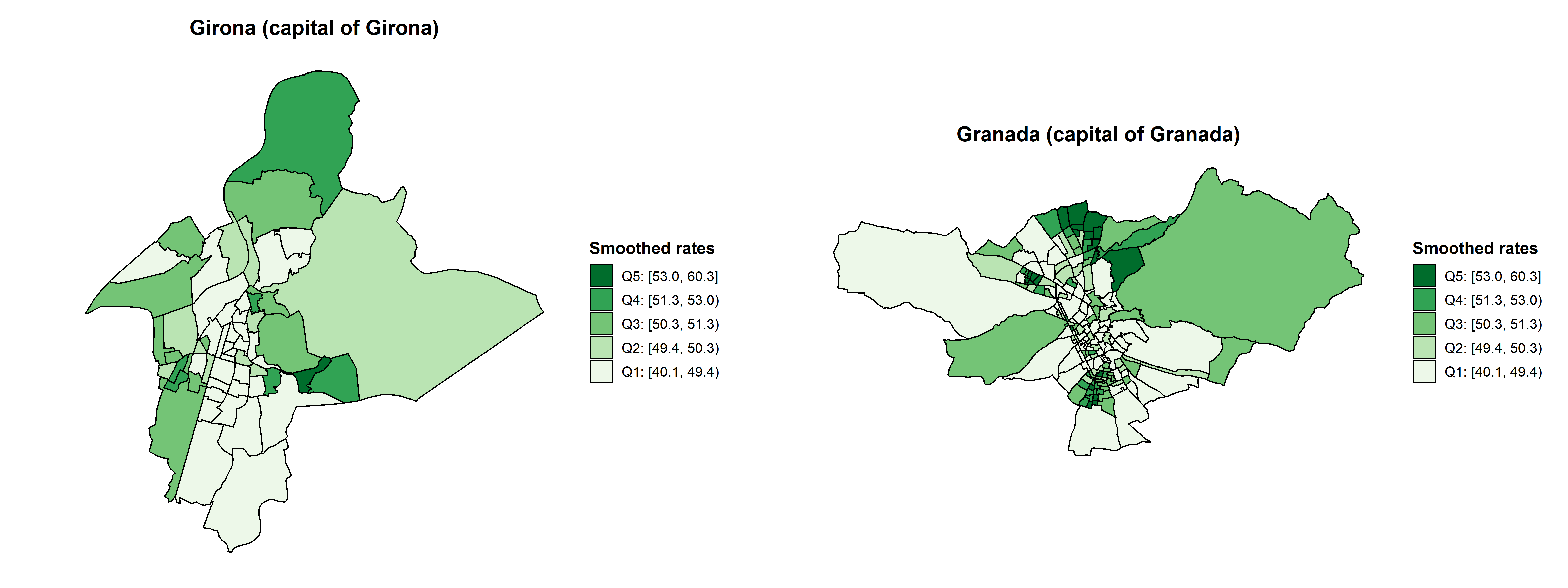

Supplement: Supplementary file 1 [file cancers-13-02820-s001.zip › SupFigure5.png]

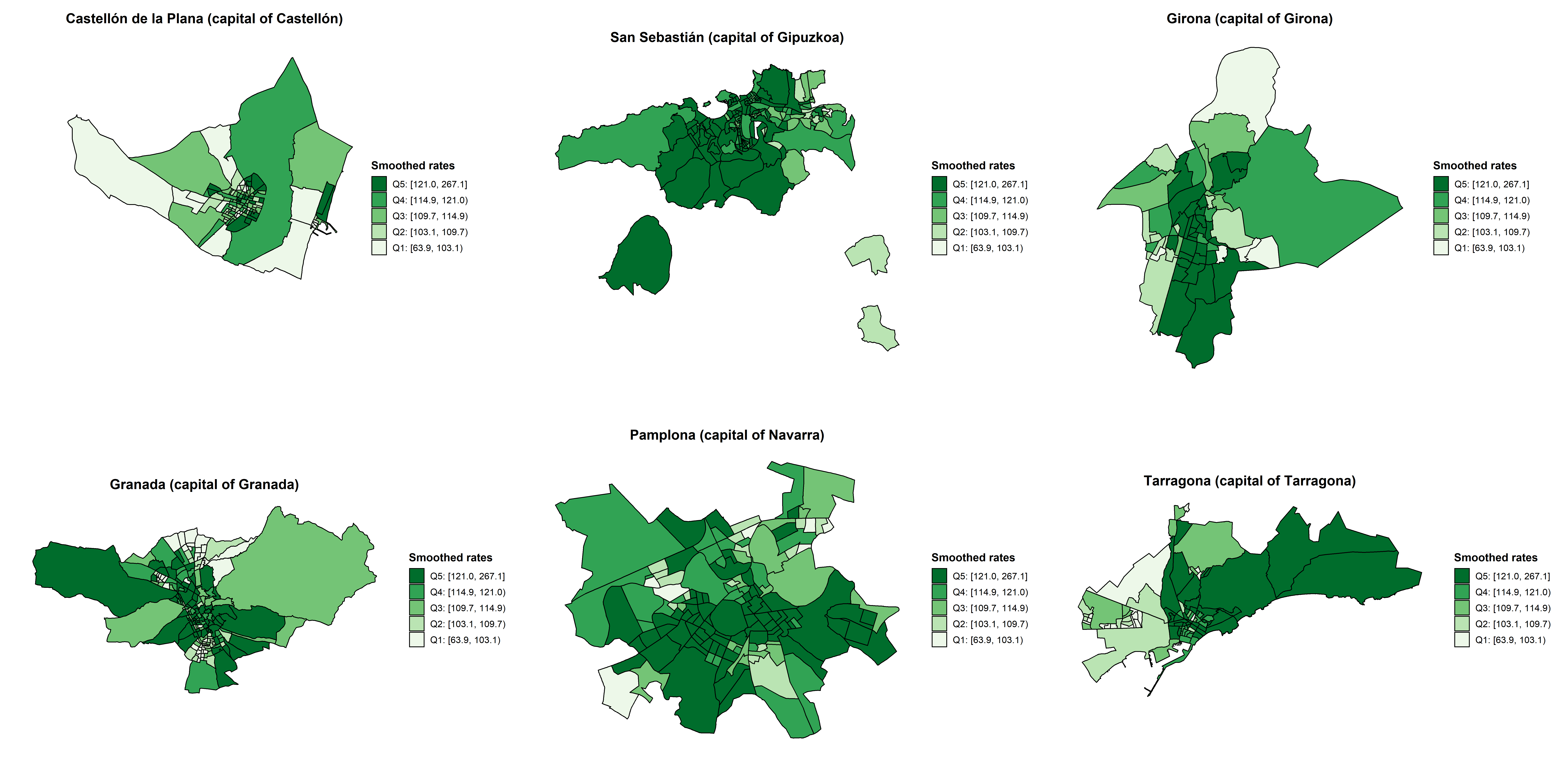

Supplement: Supplementary file 1 [file cancers-13-02820-s001.zip › SupFigure6.png]
